# Modulation of LPS-induced RAW 264.7 macrophages by *Pulsatilla koreana*-synthesized gold nanoparticles

**DOI:** 10.3389/fnut.2025.1666919

**Published:** 2025-09-18

**Authors:** Yaxi Han, Xindi Zhang, Ling Zhu, Kunlun Wang, Dixin Sha, Nina Ji, Jing Fan, Qing Chen, Kaixin Chen, Ye Zhou, Xinmiao Yao, Bo Li, Lijun Guan

**Affiliations:** ^1^Institute of Food Processing, Heilongjiang Academy of Agricultural Sciences, Harbin, China; ^2^Key Laboratory of Food Processing of Heilongjiang Province, Harbin, China; ^3^Soybean Institute, Heilongjiang Academy of Agricultural Sciences, Harbin, China

**Keywords:** *Pulsatilla koreana*, phytosynthesis, gold nanoparticle, anti-inflammation, functional food

## Abstract

Biosynthesis of gold nanoparticles using medicinal plants has emerged as a promising strategy in nanobiotechnology due to their distinctive therapeutic attributes, including biological specificity, low cytotoxicity, and inherent biocompatibility. This study presents a straightforward phytosynthetic approach that eliminates requirements for additional stabilizing agents, demonstrating exceptional process simplicity and efficiency. The formation of Pk-AuNps was confirmed by UV–Vis spectroscopy with maximum absorbance at 540 nm. Comprehensive characterization through FE-TEM, EDX, and XRD revealed spherical morphology with face-centered cubic crystalline structure, while FTIR identified critical functional groups responsible for biological activity of Pk-AuNps. DPPH radical scavenging, ABTS inhibition, and ferric reducing power analysis further revealed that Pk-AuNps possess strong antioxidant activity. Cytocompatibility evaluations in RAW264.7 and A549 cell lines revealed excellent biosafety characteristics of Pk-AuNps, highlighting their biocompatibility for potential biomedical applications. Furthermore, the anti-inflammatory properties of Pk-AuNps in LPS-stimulated murine macrophages were also investigated. Notably, Pk-AuNps demonstrated potent anti-inflammatory effects in LPS-activated macrophages, significantly attenuating pro-inflammatory mediators through dual mechanisms: (1) Inhibition of NO and PGE2 production, and (2) Downregulation of iNOS and COX-2 gene expression. These findings indicate that Pk-AuNps show promise as functional food ingredients, demonstrating multifunctional bioactive properties to developing anti-inflammatory nutraceuticals.

## Graphical abstract

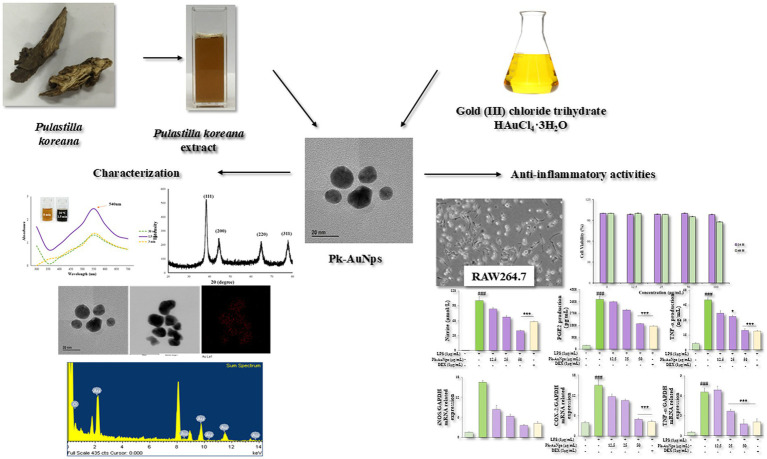

## 1 Introduction

*Pulsatilla koreana*, a perennial medicinal plant belonging to the Ranunculaceae family endemic to East Asia, has long been valued in traditional medicine and cuisine (1). Its roots are commonly consumed as functional decoctions to address ailments such as amoebic dysentery, malaria, chills, and fever (2, 3). This legacy positions it as a candidate for modern functional food development. Prior phytochemical studies have identified ranunculin (4), anemoside A, cussosaponin C (5), protoanemonin, oleanane-type triterpenoid saponins (6, 7), quinones, phenylpropanoids, and flavonoid glycosides in the both root and aerial parts of *P. koreana*. Many of these compounds are recognized bioactive constituents in functional foods.

Critically, the precise mechanism by which the plant extract governs the synthesis of stable metallic nanoparticles has been elucidated. Phytocompounds such as flavonoids, alkaloids, polyphenols, terpenoids, poly-saccharides, amino acids, organic acids, and vitamins could play key roles in reducing metal ions and stabilizing the resulting nanoparticles (8, 9). Numerous studies have associated terpenoids, a category of diverse organic polymers found in plants, with the bio-reduction of gold ions into nanoparticles. Analogously, flavonoids possess a variety of functional groups capable of reducing metal ions to nanoparticle dimensions (10).

In light of the growing emphasis on green nutritional supplements, AuNps derived from medicinal plants have gained substantial research interest. Recent studies further highlight the broad potential and applicability of plant-mediated biosynthesis of AuNps (11). For instance, Xu et al. reported the use of *Hibiscus syriacus* L. callus extract for efficient and stable AuNP synthesis, as well as the enhanced anti-inflammation efficacy (12). These advances reinforce the importance of our work within the current context of green nanotechnology and functional food development.

To the best of our knowledge, this study is the first to report the use of aqueous root extract from *P. koreana* for the phytosynthesis of Pk-AuNps. Our work advances the field in several meaningful ways: (1) by introducing a previously unexplored plant source with high reducing potential (13); (2) by establishing a rapid, energy-efficient, room-temperature synthesis protocol that eliminates the need for chemical stabilizers (14–16); and (3) by producing well-defined, quasi-spherical Pk-AuNps with a narrow size distribution (10–20 nm) and exceptional stability, as rigorously characterized by FE-TEM and UV–Vis spectroscopy. These characteristics endow the nanoparticles with strong potential for applications in biomedicinal (17) and nutraceutical (18, 19) sectors, producing particles with a narrow size distribution and exceptional stability.

In addition to synthesis, our objective is to create a cost-effective and eco-friendly synthesis method and to investigate the anti-inflammatory mechanisms of these nanoparticles in lipopolysaccharide (LPS)-induced RAW264.7 murine macrophages. Capitalizing on the traditional use of *P. koreana* in functional decoctions and its rich content of bioactive compounds, this research holds considerable promise for innovating in the areas of functional foods and nutraceutical delivery systems. For instance, Pk-AuNps could serve as anti-inflammatory additives in fortified foods or as bioavailability enhancers in advanced supplement formulations. While interdisciplinary advances continue to refine synthesis approaches, the present study focuses explicitly on the characterization and biofunctional assessment of Pk-AuNps *in vitro*. We further propose a conceptual framework that positions green synthesis nanobiotechnology at the intersection of phytochemistry, nanotechnology, and functional food science, with the ultimate goal of developing biocompatible and bioactive materials tailored for food-based applications. These include intelligent food packaging with antimicrobial and anti-inflammatory properties, and specifically, the design of next-generation functional foods aimed at promoting health through dietary means.

## 2 Materials and methods

### 2.1 Materials

Dried root segments of *Pulsatilla koreana* were obtained from the Hanbang Bio Inc., Suwon, Republic of Korea. Gold (III) Chloride Trihydrate (HAuCl_4_•3H_2_O), 2,2-Diphenyl-1-Picrylhydrazyl (DPPH), 2,2′-Azino-Bis (3-Ethylbenzothiazoline-6-Sulfonic Acid) ABTS, ascorbic acid, 3-(4,5-dimethyl-thiazol-2yl)-2, 5-diphenyl tetrazolium bromide (MTT), 2′,7′–dichlorofluorescin diacetate (DCFH-DA), dexamethasone (DEX), 4′,6-diamidino-2-phenylindole (DAPI), TRIzol reagent, and LPS were sourced from Sigma Aldrich Co. (St. Louis, MO, United States). Human lung carcinoma cell line (A549) and RAW264.7 cell line were obtained from the Korean Cell Line Bank (KCLB, Seoul, Republic of Korea). Dulbecco’s Modified Eagle Medium (DMEM), fetal bovine serum (FBS), RPMI 1640 culture medium, phosphate-buffered saline solution (PBS, pH 7.4), Dimethyl Sulfoxide (DMSO, 10%), and penicillin–streptomycin were acquired from GenDEPOT (Barker, TX, United States). The remaining chemicals employed in this study were of analytical-grade and used without further purification.

### 2.2 Preparation of *Pulsatilla koreana* extract

Segments of the *P. koreana* dried root (10 g) underwent a meticulous grinding process to achieve a fine particulate consistency. These particles were then immersed in 100 mL of sterile, distilled water. The mixture was subjected to an autoclaving process at a temperature of 100 °C for a duration of 1 h, ensuring thorough sterilization and extraction. Subsequently, the aqueous solution was carefully filtered through Whatman Grade 1 filter paper to meticulously remove any undissolved particulates, thereby yielding a homogeneous and clear extract. The filtrate was stored at 4 °C and used within 14 days to ensure stability and integrity for subsequent experiments (20).

### 2.3 Phytosynthesis of Pk-AuNps

The fabrication of gold nanoparticles was achieved through the reduction of HAuCl_4_•3H_2_O, utilized as the gold metal precursor. A 0.1 mL volume of HAuCl_4_•3H_2_O (1 mol/L) was added to 99.9 mL of an aqueous solution containing 10% *(v/v) P. koreana* extract, resulting in a final concentration of 1 mmol/L HAuCl_4_•3H_2_O in the reaction mixture. The gold reduction reaction was allowed to proceed under ambient conditions, with the formation of nanoparticles evidenced by a characteristic shift in the solution’s color to deep purple. The nanoparticles were subsequently isolated via high-speed centrifugation at 16,000 rpm for a duration of 15 min. Following centrifugation, the pallet was purified using sterile water to remove any residual impurities and then air-dried under sterile conditions overnight. The resulting powder was thus rendered suitable for further physicochemical characterizations and *in vitro* experimental applications (21, 22).

### 2.4 Characterization of Pk-AuNps

The presence and characteristic optical properties of the synthesized nanoparticles were confirmed using a Ultraviolet–Visible (UV–Vis) spectrophotometer (Ultrospec 2100 Pro, Amersham Biosciences), with the samples being scanned across a wavelength range of 300–800 nm (23). Morphological analysis, along with assessments of particle size, purity, and elemental distribution, was conducted using a high-resolution Field-Emission Transmission Electron Microscopy (FE-TEM) equipped with Energy-Dispersive X-Ray Spectroscopy (EXD) and Selected Area Electron Diffraction (SAED) (24), operated at an acceleration voltage of 200 kV (JEM-2100F, JEOL). Elemental mapping was also performed with the same instrument to further delineate the compositional uniformity across the nanoparticles.

The crystallite size of the nanoparticles was determined by X-Ray Diffraction Analysis (XRD) technique (D8 Advance, Bruker, Germany) (25) employing a Cu-Kα radiation of 1.54 Å, with the 2θ range of 20–80 ° at 6 °/min with an interval of 0.02 ° at a voltage of 40 kV and a current of 40 mA. The average nanoparticle size was calculated by Debye–Scherrer Equation, which correlates the broadening of the XRD peaks with the size of the crystalline domains:


$$D=\frac{0.9\lambda }{\beta \cos\theta }$$

where *D* is the crystallite size in nm, *λ* is the wavelength of Cu-Kα radiation in nm, β is the Full width at half maximum (FWHM) in radians, and *θ* is half of the Bragg angle in radians (26).

The hydrodynamic diameter and Polydispersity Index (PDI) of the nanoparticles were assessed using a Particle size analyzer (DLS-Photal, Otsuka Electronics, Japan) (27). This technology leverages dynamic light scattering (DLS) to probe the size distribution profiles of nanoparticles, classifying the measurements by intensity, particle number, and volume (28). The assessment was pivotal in characterizing the homogeneity and stability of the nanoparticle suspensions.

Moreover, the functional groups present on the surface of the eco-friendly synthesized Pk-AuNps were elucidated using a PerkinElmer Spectrum One Fourier Transform Infrared Spectroscopy (FTIR) spectrometer (PerkinElmer Inc., Waltham, MA, United States) covering the wave range of 4,000–450 cm^−1^ with an exquisite resolution of 4 cm^−1^ (29).

### 2.5 Antioxidant activity of Pk-AuNps

The antioxidant activity of Pk-AuNps was assessed utilizing the DPPH radical scavenging assay (30) and ABTS scavenging activity (31). We performed the DPPH assay with minor methodological adaptations to enhance the specificity of our system (32). The specific details are as follows, varying concentrations of Pk-AuNps were induced to a 0.1 mmol/L solution of DPPH solution, and the mixture was subjected to a 30 min incubation period in a dark environment to prevent photochemical interference. The samples were analyzed spectrophotometrically at 517 nm using a UV–Vis spectrophotometer.

The percentage of free radical scavenging activity was quantified using the following equation:


$$\text{Inhibition}\;(\%)=\frac{A_{\text{Control}}-A_{\text{Sample}}}{A_{\text{Control}}}\times 100$$

where *A_Control_* is the absorbance of an equivalent volume of standard methanol, and *A_Sample_* is the absorbance of the samples.

The ABTS radical scavenging activity of Pk-Nps was evaluated with slight modifications. 7.4 mmol/L ABTS solution was prepared in 2.5 mmol/L potassium persulfate. Then, the solution was incubated in the dark for 12–18 h at room temperature to obtain a stable oxidative. The stock solution of ABTS was incubated because ABTS and potassium per-sulfate reacted with each other stoichiometrically at a ratio of 0.5:1.0. This ultimately resulted in the incomplete oxidation of ABTS. However, the solution of ABTS oxidized immediately. To attain the maximal absorbance, solution must be elapsed for at least 5–6 h in the dark. Furthermore, the radical becomes stable when it is stored at room temperature in the dark for more than 2 days. To obtain an absorbance of 0.4 at 734 nm, the solution was diluted with sodium-phosphate buffer (10 mmol/L, pH 7.4). Then, 1 mL of Pk-AuNps of various concentrations (100–1,000 ppm) were mixed with the solution of ABTS. This mixed solution was incubated for 60 min in the dark. For the standard, ascorbic acid was used. Standard methanol was used as a blank. All the absorbances were noted for each sample and standard. At the wavelength 734 nm, absorbance was calculated using the same formula as DPPH radical scavenging.

### 2.6 Reducing power assay

For the measurement of the reductive ability, we tested the Fe^3+^ Fe^2+^ transformations in the presence of Pk-AuNps following the standard method (33). Different concentrations (100, 250, 500, and 1,000 μg/mL) of Pk-AuNps were mixed with 2.5 mL of phosphate buffer and 2.5 mL of (1%) potassium ferricyanide. Then, this mixture was incubated for 20 min at 50 °C and it cooled immediately. After this, 2.5 mL of 10% TCA was added to the above-mentioned solution and then the solution was centrifuged for 10 min at 3,000 rpm. Then, the collected supernatant was mixed with an equal amount of Millipore Milli-Q water. Finally, 1 mL of 0.1% ferric chloride was added to it with upper layer, and the absorbance was measured with the help of spectrophotometer at wavelength of 700 nm. Ascorbic acid was used as a standard. The percentage of reducing power was calculated using the following formula:


$$\text{Reducing power}\;(\%)=\frac{A_{\text{Control}}-A_{\text{Sample}}}{A_{\text{Control}}}\times 100$$

where *A_Control_* is the absorbance of an equivalent volume of distilled water, and *A_Sample_* is the absorbance of the samples.

### 2.7 *In vitro* biological studies of Pk-AuNps

#### 2.7.1 Cell culture

The RAW264.7 and A549 cell lines were independently cultured in DMEM and RPMI-1640 medium, respectively. Each medium was supplemented with 10% FBS and 1% Penicillin–Streptomycin to enhance cell viability and prevent microbial contamination. The cells were maintained under optimal growth conditions: at 37 °C in a humidified incubator with a 5% CO_2_ atmosphere.

#### 2.7.2 Cell viability assessment of Pk-AuNps

The *in vitro* cytotoxicity of the Pk-AuNps was tested by MTT assay (34). Cells were plated at a density of 1 × 10^4^ cells per well in 96-well culture plates (Corning Costar, Lowell, NY, United States) and incubated overnight to allow for attachment. Upon completion of the incubation period, the cells underwent a 24 h treatment regimen with the nanoparticles, with three wells free of Pk-AuNps used as controls. Subsequently, 20 μL of MTT solution (5 mg/mL, in PBS) was introduced to each well for a 3 h interval. The spent media and MTT reagent were then aspirated and superseded with 100 μL of DMSO (10%), allowing an additional 30 min for incubation in dark and shaken. The amount of formazan, an indicator of viable cells, was quantified via a multi-mode microplate reader (Bio-Tek Instruments, Winooski, VT), using test and reference wavelengths of 570 nm and 630 nm, respectively.

#### 2.7.3 Measurement of NO, PGE2, and TNF-α production

The murine macrophage RAW264.7 cells were co-treated with 1 μg/mL LPS and a dose of Pk-AuNps and then subjected to a 24 h incubation period. The supernatant was collected for further analysis. To detect the level of nitric oxide (NO), an equal volume of Griess reagent was combined with 100 μL of the harvested culture supernatant. This mixture was then assessed using a multi-plate reader, set to a wavelength of 540 nm. The levels of Prostaglandin E2 (PGE2) and Tumer Necrosis Factor-α (TNF-α) were determined through Enzyme-Linked Immunosorbent Assay (ELISA) commercially available kits strictly following the manufacturer’s procedures (R&D Systems, Minneapolis, MN, United States) (20).

#### 2.7.4 ROS generation

The capacity of Pk-AuNps to provoke oxidative stress was gaged using the DCFH-DA assay (35). Adherent RAW264.7 cells, following being seeded in a 96-well black/clear bottom plate (Corning Costar, Lowell, NY, United States), were exposed to varied concentrations of Pk-AuNps for 24 h. Subsequently, 100 μL of DCFH-DA reagent, prepared at a concentration of 15 μmol/L, was added and permitted to incubate for 30 min under low-light conditions. Thereupon, the fluorescence, a measure of reactive oxygen species (ROS) generated within the cells, was read using a Synergy™2 microplate reader (Bio-Tek Instruments, Winooski, VT).

#### 2.7.5 Gene expression studies

RAW264.7 macrophages were seeded at a density of 1 × 10^6^ cells/well in a 6-well culture plate (Corning Costar, Lowell, NY, United States). After an initial overnight incubation, 24 h of treatment with or without diverse doses of Pk-AuNps in the presence or absence of LPS stimulation was added. Total RNA was extracted using the TRIzol reagent. The cDNA synthesis was conducted in accordance with the supplier’s instructions (Thermo Scientific, EU, Lithuania). qPCR was carried out using the primers shown in Table 1 (20). The relative gene expression levels were normalized to the amount of *GAPDH*. mRNA by the ΔCt method (36).

**Table 1:** Primer sequences used for gene expression analysis by qPCR.

Primer	Sequence
*iNOS*	Forward: 5′-GTG GTG ACA ACG ACA TTT GG-3′
	Reverse: 5′-GGC TGG ACT TTT CAC TCT GC-3′
*COX-2*	Forward: 5′-GGA TGC GCT GAA ACG TGG A-3′
	Reverse: 5′-CAG GAA TGA GTA CAC GAA GCC-3′
*TNF-α*	Forward: 5′-AGT CCG GGC AGG TCT ACT TT-3′
	Reverse: 5′-GCA CCT CAG GGA AGA GTC TG-3′
*GAPDH*	Forward: 5′-CAA GGT CAT CCA TGA CAA CTT TG-3′
	Reverse: 5′-GTC CAC CAC CCT GTT GCT GTA G-3′

#### 2.7.6 Immunofluorescence staining

RAW264.7 cells were cultured overnight on 8-well chamber slides (SPL Life Sciences Co., Ltd., Korea). The cells were pre-conditioned with Pk-AuNps for 2 h to evaluate their potential modulatory effects, followed by a stimulation phase with LPS (1 μg/mL) for an additional 2 h. Subsequently, the cells were meticulously washed with PBS, then fixed with a 3.7% formaldehyde solution to preserve cellular morphology and permeabilized with 0.5% Triton X-100 for 10 min to facilitate antibody penetration. Followed, the slides were incubated overnight at 4 °C with rabbit monoclonal anti-NF-κB p65 antibodies (1:100 dilution; Santa Cruz Biotechnology, Santa Cruz, CA, United States). After thorough washing to remove unbound primary antibodies, the slides were incubated in the dark for 1 h with Alexa Fluor 488-conjugated goat anti-rabbit IgG secondary antibodies (1:200; Cell Signaling Technology, Beverly, MA, United States) to detect the primary antibody binding. For nuclear counterstaining, the slides were performed using DAPI, a fluorescent stain, at a concentration of 10 mg/mL for 15 min, allowing for the visualization of cellular nuclei. Finally, the immunofluorescence-stained slides were examined using an inverted research fluorescence microscope, capturing images that reveal the intracellular localization of NF-κB p65 in response to the experimental treatments (37–39).

### 2.8 Statistical analysis

Experimental trials were performed in triplicate to ensure their accuracy and reliability. The resultant data are presented as the mean value ± SD value. For the identification of significant differences among groups, the *p* value threshold was set at *p* ≤ 0.05. Statistical evaluations were facilitated by employing GraphPad Prism software version 9.03 (GraphPad Software Inc., La Jolla, CA). The significance of discrepancies between observed values was ascertained using a one-way ANOVA.

## 3 Results and discussions

### 3.1 Phytosynthesis of gold nanoparticles

The green synthesis of gold nanoparticles using *P. koreana* root extract represents a novel and environmentally sustainable approach within the field of nanobiotechnology. Our method is particularly notable for its rapid reduction of gold salt at room temperature, as demonstrateded by the swift color change observable within seconds, underscoring the high reactivity and efficiency of the bio-reduction process.

**Figure 1:**
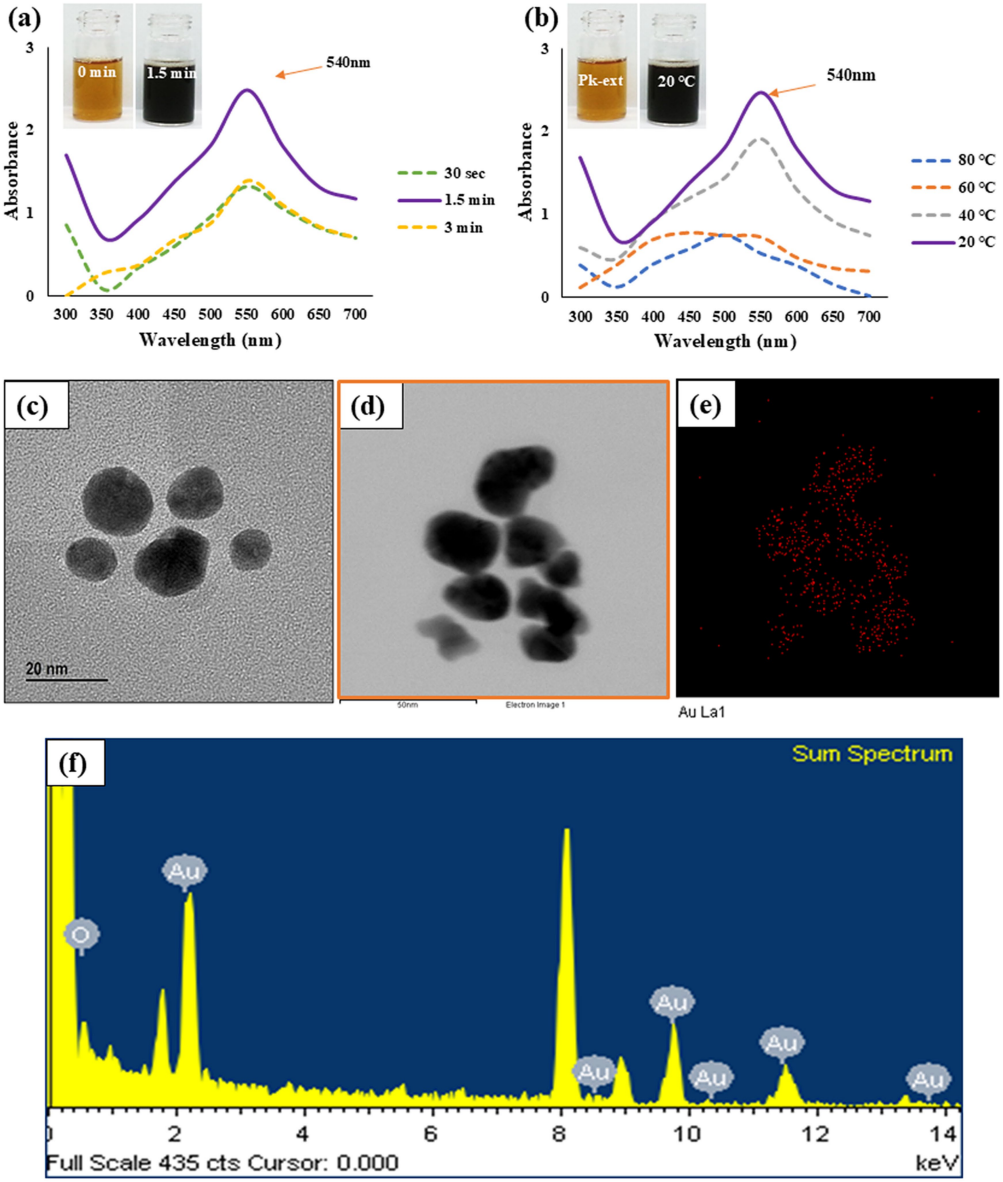
Time **(a)** and temperature **(b)** dependent UV–Vis spectrometry demonstrates the formation of Pk-AuNps. FE-TEM **(c)**, elemental mapping **(d,e)**, and EDX spectrum **(f)** exposit the morphology and chemical properties of Pk-AuNps.

The formation of Pk-AuNps was characterized by a profound visual indicator—a deep purple coloration that emerged almost instantaneously, insets from Figures 1a,b (23). This rapid color change indicates not only the surface plasmon resonance (SPR) but also the excitation of free electrons by incident light, which induces oscillations at the frequency of visible light wavelengths. Furthermore, this phenomenon serves as evidence of the innovative and rapid reduction capability of the *P. koreana* root extract, which is a distinctive feature of our study.

Additionally, following a 30-s incubation period, the UV–Vis spectrum of Pk-AuNPs exhibited a maximum absorption peak at 540 nm (Figure 1). This wavelength (λmax) is characteristic of spherical gold nanoparticles, thereby confirming the successful synthesis of the particles (40). No further increase in absorption was detected after this period. The rapid emergence and intensity of this plasmonic peak attest to the high efficiency of the proposed synthesis route. Similar λmax values have been previously documented for AuNps synthesized using biological extracts, as reported by Elavazhagan and Arunachalam (41).

Under the standard reaction conditions employing 10% (*v/v*) *P. koreana* root extract and 1 mmol/L gold salt in a 100 mL system, the yield of gold nanoparticles was determined to be 16.72 ± 0.39 mg per 100 mL reaction mixture.

Furthermore, the biosynthesis of Pk-AuNps was evaluated across a temperature range from 20 to 80 °C (Figure 1b). Although absorption intensity increased with temperature, the sharpest and most defined plasmon bands were consistently observed at 20 °C, suggesting optimal nanoparticle formation under mild conditions. Finally, the stability of the biosynthesized nanoparticles was assessed over a seven-day period at room temperature. UV–Vis spectrophotometric analysis revealed consistent absorption values over time, confirming the long-term stability of the Pk-AuNps.

### 3.2 Characterization of Pk-AuNps

#### 3.2.1 FE-TEM analysis

Employing FE-TEM, we conducted a comprehensive investigation to elucidate the characteristics and dimensionality of the synthesized nanostructures (42), with a particular focus on gold nanoparticles. Our study uniquely demonstrates that the Pk-AuNps predominantly exhibit a quasi-spherical configuration, with sizes precisely ranging from 10 to 20 nm, as clearly portrayed in Figures 1c,d. The controlled synthesis of these nanoparticles achieved by modulating key parameters such as reaction temperature, and incubation time can be readily extended to the tailored production of other gold nanoparticles. This is accomplished by leveraging the same bio-reduction principles mediated by the phytochemical constituents present in the *P. koreana* root extract, which act as both reducing and stabilizing agents. Such reproducibility and tunability represent an innovative contribution to the field of green nanobiotechnology.

Elemental mapping of the nanoparticles provided a vivid electron imagery, confirming the elemental uniformity across the particles, as depicted in Figures 1d,e. This uniformity is essential for the consistent performance of Pk-AuNps in various applications.

The purity of these metallic nanoparticles was further authenticated through meticulous examination of the EXD spectra, ensuring the high-quality material needed for advanced nanodevices (43). The distinct peaks at 2.15 keV, corresponding to the characteristic absorption edges of metallic gold, were observed in Figure 1f, indicating the exceptional purity of Pk-AuNps. The peak recorded at 8 keV correspond to coper grid used for analysis.

The ability to control the crystallographic facets is pivotal for tailoring the electronic properties of gold nanoparticles, which is an innovative aspect of our methodology, highlighting the potential for their use in nanoscale devices with specific performance criteria.

#### 3.2.2 XRD analysis

**Figure 2:**
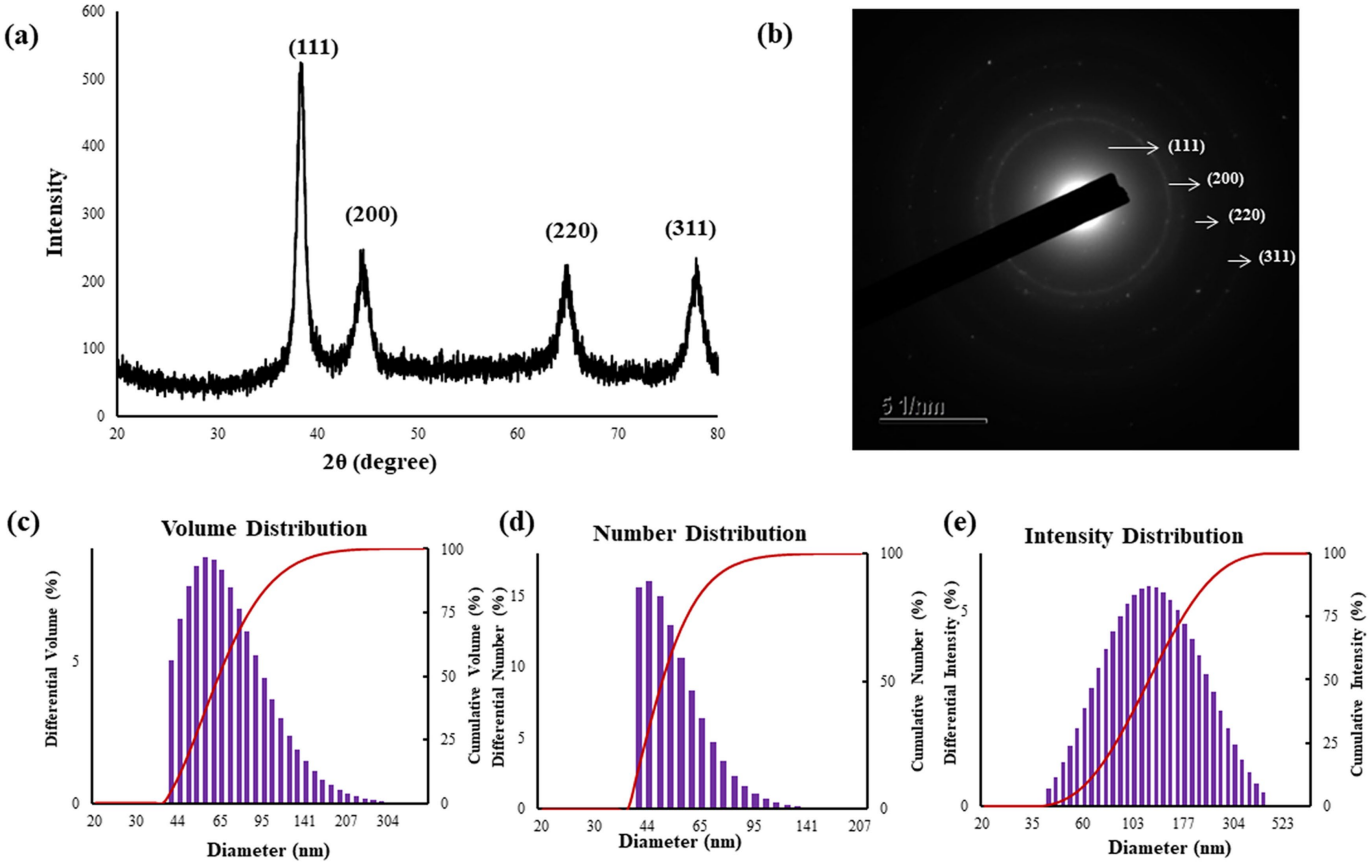
XRD analysis **(a)** confirms the crystallinity of the Pk-AuNps, SAED patterns **(b)**, and particle size distributions of Pk-AuNps respective to volume **(c)**, number **(d)**, and intensity **(e)**.

The XRD pattern of the crystalline Pk-AuNps, with its pronounced and intense diffraction peaks as highlighted in Figure 2a, is indicative of a well-defined crystallographic structure, which is a prerequisite for various high-performance applications (44).

The four characteristic diffraction peaks, indexed to the [111], [200], [220], and [311] lattice, and primarily composed of [111] orientation, align with Bragg’s law and the reference patterns of the Joint Committee on Powder Diffraction Standards (JCPDS No. 04–0784). This precise peak assignment confirms the face-centered cubic (FCC) structure of the gold nanoparticles and reflects a high degree of crystallographic ordering, essential for their robust physical and chemical properties (45, 46). The average diameter of nanoparticles was estimated by Debye–Scherrer equation: Pk-AuNps maintained average crystallite sizes of 6.31 nm. The SAED patterns likewise confirmed the polycrystalline nature of the nanoparticles (47), as shown in Figure 2b.

In addition to the applications in catalysis, sensing, and electronics, the high crystallinity and biocompatibility of these nanoparticles also make them suitable candidates for innovative uses in the food science industry. For instance, the Pk-AuNps could be integrated into active food packaging to enhance the shelf life of perishable goods by preventing microbial contamination. Their inherent antimicrobial properties can be harnessed to create self-sanitizing surfaces that maintain food safety and quality.

Furthermore, the gold nanoparticles’ non-toxic nature and ability to withstand high temperatures suggest their potential suitability for use in food processing environments, where they could contribute to thermal management applications. The innovative synthesis method employed in our study ensures the formation of highly crystalline nanoparticles with a narrow size distribution, which is evident from the sharp and intense peaks in the XRD pattern. This controlled synthesis is essential for achieving the uniformity expected in food-grade nanomaterials—a critical prerequisite for any future functional applications. It should be noted, however, that the actual performance and stability of these nanoparticles under realistic food processing conditions—such as exposure to thermal cycling, shear forces, or variable pH—have not been evaluated in this study and warrant further investigation.

#### 3.2.3 Size distribution measurements

The size distribution profile of the biosynthesized Pk-AuNps was meticulously characterized using DLS, a technique that assesses particle size with respect to volume, number, and intensity, as depicted in Figures 2c–e. The resulting size distribution histogram exposed a moderately polydisperse population, with a Z-average value of 244.8 nm for Pk-AuNps, accompanied by PDI of 0.229. This observation indicates that the nanoparticles produced through the green synthesis process using *P. koreana* are not monodisperse but exhibit a common distribution type for biological syntheses.

The primary and secondary metabolites of *P. koreana*, including ranunculin, anemoside A, phenylpropanoids, and flavonoid glycosides, could potentially contribute to the formation of a protective capping layer around the metallic nanoparticles, thereby preventing their agglomeration (6). The discrepancy between the average sizes of the biogenic nanoparticles determined by XRD and FE-TEM versus DLS can be elucidated by the distinct measurement principles and sample states inherent to each technique. XRD anticipates the crystallite size of the nanoparticles in their dried state, providing an insight into the primary particles’ dimensions. FE-TEM further provides two-dimensional projection measurements of individual particles or small agglomerates under high vacuum on dried specimens. In contrast, DLS measures the hydrodynamic diameter in an aqueous suspension. This hydrodynamic diameter encompasses the metallic core, tightly bound hydration layers, and the biomolecular corona derived from the plant metabolites stabilizing the nanoparticles (48). Crucially, DLS exhibits high sensitivity toward any aggregates or agglomerates present in the suspension, with its intensity-weighted distribution heavily influenced by larger species. This phenomenon explains the observed difference where XRD and FE-TEM report the core or near-core dimensions, while DLS reflects the larger hydrodynamic size including the capping layer and potential aggregates in solution. Therefore, this disparity underscores the importance of employing multiple complementary characterization techniques to fully understand the size distribution, surface characteristics, and the interaction of synthesized nanoparticles with their surrounding environment (49, 50).

Moreover, the FE-TEM images corroborated the DLS findings by revealing a range of sizes within the nanoparticle population, confirming the moderate polydispersity. The acknowledgment of this moderate polydispersity is crucial for applications requiring a tailored size distribution, such as nutrient delivery or sensing applications, where nanoparticle interactions with biological systems are influenced by their size and distribution characteristics (51).

#### 3.2.4 FTIR spectroscopic analysis

**Figure 3:**
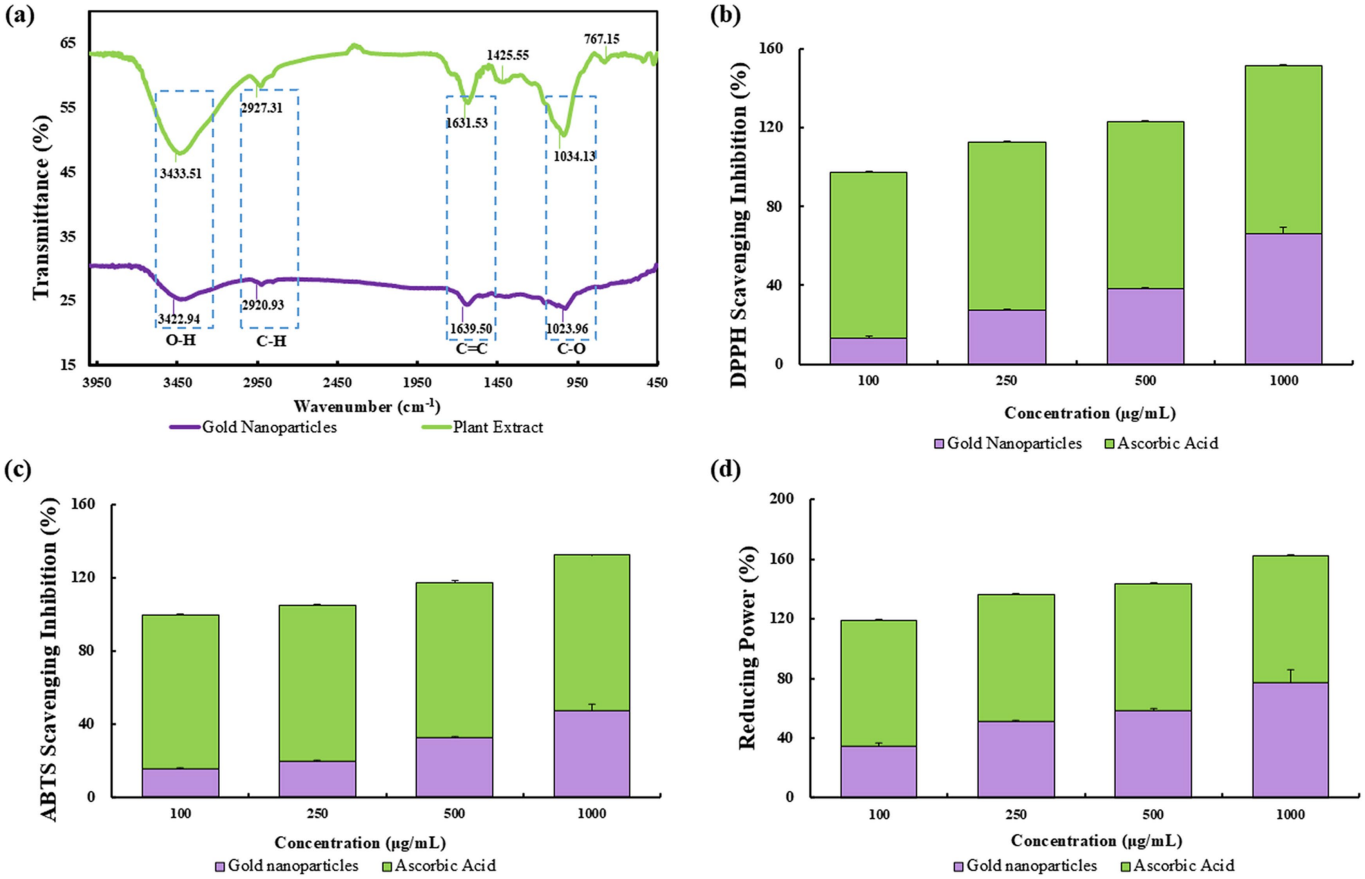
FTIR spectra of *P. koreana* root extract and Pk-AuNps **(a)**. The antioxidant activity comparison analyzed by DPPH **(b)**, ABTS **(c)**, and reducing power **(d)**.

Employing FTIR, we characterized the organic molecules on the surface of bio-reduced gold nanoparticles synthesized from *P. koreana*. The FTIR spectra disclosed a distinctive absorption pattern that corroborated the presence of specific functional groups on the nanoparticle surface, as depicted in Figure 3a. Notably, the bands at 3364–3345 cm^−1^ corresponded to O–H stretching vibrations (52), indicative of hydroxyl groups, while the regions of 1,626–1,634 cm^−1^ and 1,016–1,067 cm^−1^ were attributed to C–H and C═C/C–O groups (53), respectively, reflecting the presence of methylene/aromatic and alkene/ether functionalities. These groups are crucial for the stabilization of Pk-AuNps and are suggested to enhance their biological activity through surface capping. The disappearance of FTIR bands at 1425.55 cm^−1^ and 767.15 cm^−1^ in Pk-AuNps, compared to the *P. koreana* extract, originated from synthesis-driven chemical transformations and surface interactions (54). The 1425.55 cm^−1^ band, characteristic of aliphatic C-H bending vibrations or −COO^−^ symmetric stretching, vanished due to vibration restriction from hydrophobic adsorption onto Pk-AuNP surfaces or -COOH deprotonation forming coordinating carboxylates. The 767.15 cm^−1^ band, indicative of aromatic C-H out-of-plane bending in polyphenols, disappeared owing to oxidative aromatic ring degradation or hydrogen-bond-mediated peak broadening/redshift. Concomitant loss of these bands confirms polyphenols mediated metal ion reduction through aromatic electron donation and nanoparticle stabilization via aliphatic chain adsorption or carboxylate coordination. Enhanced spectral changes from purification-induced removal of unbound molecules provide spectroscopic evidence for plant metabolites’ role in Pk-AuNP synthesis (55).

The findings from the FTIR spectra underscore the potential of Pk-AuNps as a nano-carrier platform. The surface composition characterized by FTIR analysis offers a basis for optimizing the biological performance of these nanoparticles (56, 57).

### 3.3 Assessment of antioxidant ability

Free radicals, characterized by an unpaired number of electrons, are highly reactive species that can be generated through the interaction of oxygen with certain molecules (58, 59). In the context of our study, the antioxidant activity of Pk-AuNps was evaluated through DPPH free radicals scavenging, ABTS assay and reducing power assay, as illustrated in Figures 3b–d.

The DPPH assay is a de facto method for assessing free radical scavenging capacity, providing a straightforward visual and quantitative measure of antioxidant potential. The IC_50_ values, determined through linear regression analysis, indicated a concentration of 668.0 μg/mL for Pk-AuNps, which demonstrated a dosage-dependent free radical scavenging effect, consistent with the typical behavior expected of antioxidants (shown in Figure 3b). This observed activity is likely attributed to specific bioactive compounds present in the plant extract, particularly *Pulsatilla* saponins (57) and flavonoids (60), both of which are well-documented in the literature for their intrinsic antioxidant properties. While the extract contains a complex mixture of phytochemicals, these two groups of compounds are proposed as prominent contributors to the antioxidant effects based on their known high reactivity and previously established mechanisms (61).

The ABTS radical cation decolorization assay, as illustrated in Figure 3c, demonstrated a positive correlation between the concentration of Pk-AuNps and the scavenging activity against ABTS radicals. Specifically, the highest concentration tested, 1,000 μg/mL, yielded a maximum inhibition of 47.34%, indicating a dose-dependent enhancement in the antioxidant capacity of Pk-AuNps.

The reducing power assay, as depicted in Figure 3d, was conducted using varying concentrations (100, 250, 500, and 1,000 μg/mL) of Pk-AuNps. Notably, the highest concentration of 1,000 μg/mL exhibited a remarkable reducing power scavenging activity of 77.46%. This suggests that Pk-AuNps possess a significant capacity to facilitate the reduction of ferric ions, thereby augmenting the overall antioxidant activity (62). The mechanism underlying this phenomenon is believed to be the electron-donating ability of Pk-AuNps, which can effectively neutralize free radicals and stabilize them, thus preventing oxidative damage to biological systems.

These findings imply that flavonoids, quinones, and phenols, which form the protective capping layer of Pk-AuNps, appear to be the main sources of the free radical scavenging activity. There have been reports of the antioxidant properties of biosynthetic nanoparticles made from an aqueous extract of the leaves of *Hagenia abyssinica*, *Carissa carandas*, and *Clerodendrum inerme* (19). The antioxidant efficacy of AuNps produced by the aqueous extract of *P. koreana* has never been reported before. This green synthesis is cost-effective, environmentally benign, and helps create novel and less expensive antioxidant agents for biomedicine.

### 3.4 *In vitro* biological studies of Pk-AuNps

#### 3.4.1 Cell viability

**Figure 4:**
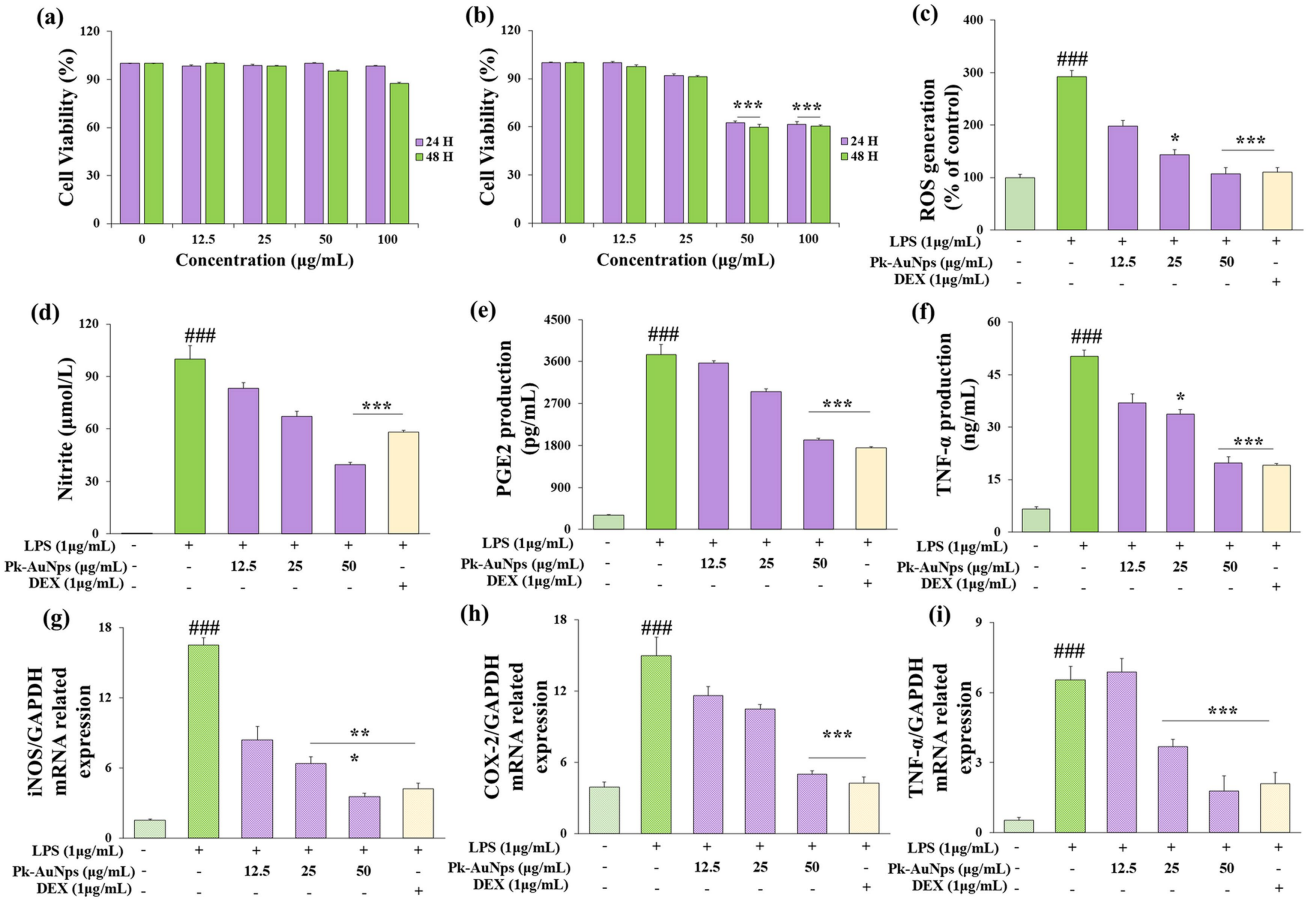
Evaluation of the cell viability in RAW 264.7 **(a)** and A549 **(b)** cells, 24 h and 48 h after Pk-AuNps treated. Effects of Pk-AuNps on LPS-induced ROS production **(c)**. Determination of the reduction of nitrite **(d)**, PGE2 **(e)** and TNF-α **(f)** release. Expression of *iNOS* **(g)**, *COX-2* **(h)** and *TNF-α* **(i)** in gene level. The values are presented as means ± SD of three independent experiments. ^###^*p* ≤ 0.001 for the LPS treated group compared with the control group. *p ≤ 0.05 and ***p ≤ 0.001 compared with the LPS treated group.

In this current study, RAW264.7 and A549 cells were used as model systems to assess the cytotoxicity of Pk-AuNps. Cell viability was measured *in vitro* using the MTT assay. Cells were treated with Pk-AuNps at concentrations ranging from 1 to 100 μg/mL for 24 or 48 h. Results showed no significant cytotoxicity in RAW264.7 cells even at the highest concentration tested (50 μg/mL) after 24 h, well, at 48 h in RAW264.7 cells, 100 μg/mL Pk-AuNps showed minimal cytotoxicity (Figure 4a). In contrast, the A549 cell line exhibited significant growth inhibition at 50 μg/mL (Figure 4b). These findings enabled the selection of 50 μg/mL as the maximum concentration for subsequent *in vitro* experiments.

#### 3.4.2 *In vitro* anti-inflammatory effect of Pk-AuNps

Macrophages constitute one of the key immune defense mechanisms within the human body endowed with the ability of phagocytosis (63). They are also involved in the inflammatory response by generating both inducible nitric oxide synthase (iNOS) and cyclooxygenase-2 (COX-2) through NF-κB activation (2, 64). Previous studies have reported that NO production by iNOS and PGE2 was derived from COX-2 and played an essential role during the inflammatory reaction process. Antigen-presenting cells (APCs), such as macrophages, play a vital role in initiating immune responses (65, 66). The complement system also contributes to the inflammatory process.

﻿We first verified the antioxidant capacity of Pk-AuNps. As shown in Figure 4c, the ROS level decreased in a concentration-dependent manner with increasing concentrations of Pk-AuNPs. At a treatment concentration of 50 μg/mL, the cellular ROS level was significantly reduced. This finding laid the foundation for subsequent research on anti-inflammatory activity. Consequently, to ascertain the anti-inflammatory effect of Pk-AuNps, the levels of NO, TNF-α, and PGE2 were measured. We also undertook the determination of the production of NO, TNF-α, and PGE2 in LPS-induced RAW264.7 cells, with or without Pk-AuNps treatment, using DEX as a positive control.

In Figure 4d, our results indicated an increase in NO levels in the LPS-induced RAW264.7 cells compared with the basal level without LPS (1 μg/mL) treatment. The dose-dependent treatment of Pk-AuNps significantly reduced the NO level. The qPCR analysis in Figure 4g exhibited that the expression of *iNOS* at the gene level was also suppressed. Figure 4d indicated that the LPS-induced PGE2 production release was decreased by treating with Pk-AuNps in a dose-dependent manner. In addition, the gene expression of *COX-2* significantly decreased with the Pk-AuNps treatment of the LPS-stimulated murine macrophages (Figure 4h). Consequently, Pk-AuNps blocked the activities of both *iNOS* and *COX-2* at the mRNA level in the LPS-induced RAW264.7 cells.

In previous research, the extract of *P. koreana* was found to suppress NO production in LPS-stimulated RAW264.7 cells. Moreover, these oleanane-type triterpenoid saponins showed a considerable degree of activity in inhibiting TNF-α-induced NF-κB activation (64). Notably, Pk-AuNps could significantly inhibit the PGE2 levels in 25 μg/mL (Figure 4e). Activated macrophages and T cells are capable of generating TNF-α and other proinflammatory cytokines as an integral part of the immune response (67, 68). By conducting ELISA to test the production (17) of TNF-α in LPS-induced RAW264.7 cells, we evaluated the efficacy of Pk-AuNps in diminishing proinflammatory mediators. Pk-AuNps reduced the LPS-induced release of TNF-α in a dosage-dependent manner, as depicted in Figure 4e. Then, qPCR was employed to assess the *TNF-α* gene level. It was observed that TNF-α levels in the stimulated RAW264.7 cells were reduced by Pk-AuNps (Figures 4f,i). The attenuating of TNF-α in our study implied that Pk-AuNps might possess anti-inflammatory attributes (69).

**Figure 5:**
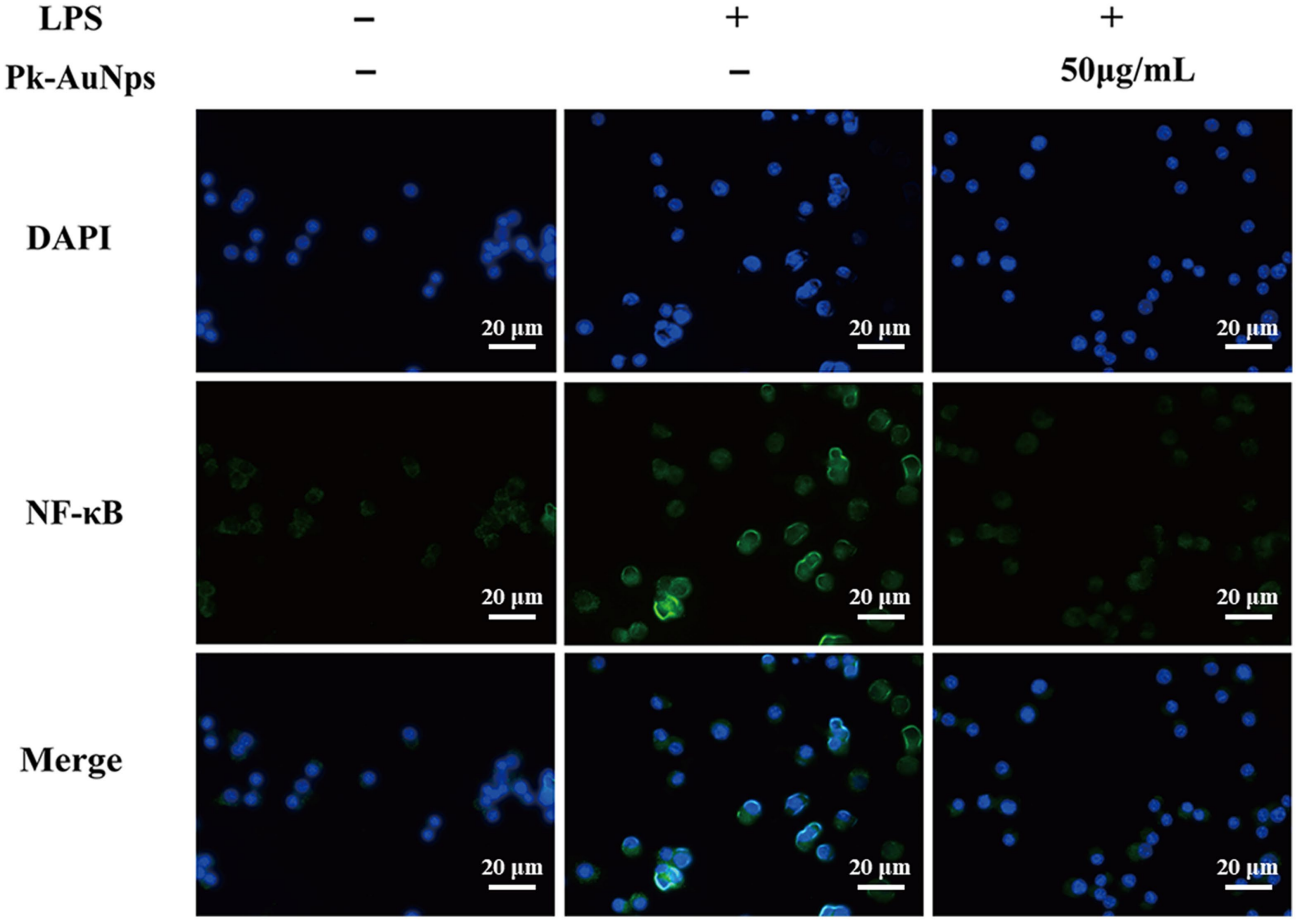
Immunofluorescence staining of NF-κB p65 expression in RAW 264.7 macrophages.

According to the immunofluorescence staining presented in Figure 5, the density of NF-κB (green fluorescence) was conspicuously augmented at 2 h of LPS exposure. Nevertheless, LPS-induced nuclear translocation was markedly inhibited by the co-treatment of Pk-AuNps, the quantities of nuclear NF-κB p65 were escalated following LPS exposure, yet Pk-AuNps significantly impeded this LPS-induced nuclear translocation. These findings align with the concept of immunomodulation, suggesting that Pk-AuNps may have potential in regulating immune responses (64) and inflammatory conditions. Further studies could explore its interaction with immune checkpoints and cytokine networks for a comprehensive understanding of its immunological effects.

Within the framework of the immune system, cytokines such as interleukin-1β (IL-1β), interleukin-6 (IL-6), and interferon-γ (IFN-γ) play pivotal roles in regulating inflammatory responses. The major histocompatibility complex (MHC) molecules are indispensable for antigen presentation (70). Regulatory T cells contribute significantly to maintaining immune homeostasis (71). The complement cascade constitutes another crucial element of the immune defense mechanism (72). Future studies could explore the interaction of Pk-AuNps with these immune factors to furnish a more comprehensive comprehension of its immunomodulatory effects.

## 4 Conclusion

The aqueous extract of *P. koreana* showed remarkable efficacy in the synthesis of gold nanoparticles (Pk-AuNps), as initially evidenced by the characteristic color transition from yellow to purple. UV–vis spectral analysis revealed a distinct SPR peak at 540 nm, confirming nanoparticles formation.

FE-TEM revealed spherical nanoparticles with sizes ranging from 5 to 20 nm (average nanoparticle size: 11.11 ± 3.5 nm). XRD confirmed the crystalline nature of the material. FTIR spectroscopy enabled the identification of the principal functional groups in the plant extract and the synthesized Pk-AuNps. The high stability of nanoparticles in the colloidal solution was verified by DLS characterizations. Biological activities, encompassing antioxidant, as well as *in vitro* cytotoxic efficacy, and anti-inflammatory properties, were investigated and determined to be dose-dependent. The Pk-AuNps demonstrated elevated antioxidant activity and target-oriented cytotoxicity toward cancer cell line (in contrast to negligible cytotoxicity against normal cell line). *In vitro* investigations disclosed that Pk-AuNps exhibited a superior cellular anti-inflammatory potential in RAW264.7 cells by suppressing NO, TNF-*α*, PGE2, and associated gene expression. Overall, the extract of *P. koreana* exhibits significant potential for the generation of multi-functional AuNps, presenting prospects for various biomedical applications. Future orientations could incorporate their application in treatment of chronic inflammatory disorders such as rheumatoid arthritis and inflammatory bowel diseases. They might also be utilized in modulating immune responses in autoimmune conditions or serve as adjuncts in post-operative care to prevent inflammation-related complications. Furthermore, the exploration of their combined use with existing anti-inflammatory drugs to enhance therapeutic outcomes and minimize side effects holds considerable promise.

## Data Availability

The original contributions presented in the study are included in the article/supplementary material, further inquiries can be directed to the corresponding author.
